# LncRNA ILF3‐AS1 mediates oxidative stress and inflammation through miR‐504‐3p/HMGB1 axis in a cellular model of temporal lobe epilepsy

**DOI:** 10.1002/brb3.3615

**Published:** 2024-08-12

**Authors:** Peipei Gao, Ying Wu, Zhixin Yan

**Affiliations:** ^1^ Department of Pediatrics Cangzhou Central Hospital Cangzhou Hebei Province People's Republic of China

**Keywords:** ILF3‐AS1, inflammatory response, mir‐504‐3p/HMGB1 axis, oxidative stress, temporal lobe epilepsy

## Abstract

**Background:**

Temporal lobe epilepsy (TLE), a prevalent neurological disorder, is associated with hippocampal oxidative stress and inflammation. A recent study reveals that the long noncoding RNA ILF3 divergent transcript (ILF3‐AS1) level is elevated in the hippocampus of TLE patients; however, the functional roles of ILF3‐AS1 in TLE and underlying mechanisms deserve further investigation. Hence, this study aimed to elucidate whether ILF3‐AS1 is involved in the pathogenesis of TLE by regulating oxidative stress and inflammation and to explore its underlying mechanism in vitro.

**Methods:**

Human hippocampal neurons were subjected to a magnesium‐free (Mg^2+^‐free) solution to establish an in vitro model of TLE. The potential binding sites between ILF3‐AS1 and miRNA were predicted by TargetScan/Starbase and confirmed by dual luciferase reporter assay. Cell viability and damage were assessed by cell counting kit‐8 and lactate dehydrogenase assay kits, respectively. Levels of reactive oxygen species, malondialdehyde, and superoxide dismutase were determined by commercial kits. Levels of Interleukin‐6 (IL‐6), IL‐1β, and tumor necrosis factor‐alpha were quantified by enzyme‐linked immunosorbent assay. The expressions of gene and protein were determined by quantitative real‐time polymerase chain reaction and Western blot analysis.

**Results:**

In Mg^2+^‐free‐treated hippocampal neurons, both ILF3‐AS1 and HMGB1 were highly up‐regulated, whereas miR‐504‐3p was down‐regulated. ILF3‐AS1 knockdown ameliorated Mg^2+^‐free‐induced cellular damage, oxidative stress, and inflammatory response. Bioinformatics analysis revealed that miR‐504‐3p was a target of ILF3‐AS1 and was negatively regulated by ILF3‐AS1. MiR‐504‐3p inhibitor blocked the protection of ILF3‐AS1 knockdown against Mg^2+^‐free‐induced neuronal injury. Further analysis presented that ILF3‐AS1 regulated HMGB1 expression by sponging miR‐504‐3p. Moreover, HMGB1 overexpression reversed the protective functions of ILF3‐AS1 knockdown.

**Conclusion:**

Our findings indicate that ILF3‐AS1 contributes to Mg^2+^‐free‐induced hippocampal neuron injuries, oxidative stress, and inflammation by targeting the miR‐504‐3p/HMGB1 axis. These results provide a novel mechanistic understanding of ILF3‐AS1 in TLE and suggest potential therapeutic targets for the treatment of epilepsy.

## Graphical abstract

Our findings indicate that ILF3‐AS1 contributes to hippocampal neuron injuries following temporal lobe epilepsy by inhibiting neuronal oxidative stress and inflammation through the miR‐504‐3p/HMGB1 pathway in vitro.

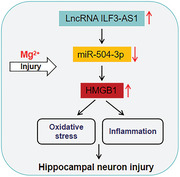

## 1 INTRODUCTION

Temporal lobe epilepsy (TLE), a common subtype of epilepsy, is a complex and heterogeneous disease that presents significant challenges to both patients and healthcare professionals (Cascino, 2009). The hallmark feature of TLE is seizures originating in the temporal lobe and potentially spreading to surrounding brain regions (Bartolomei et al., 2008). The causes of TLE are multifactorial, encompassing various factors like genetic predisposition, traumatic brain injury (TBI), encephalitis, and brain tumors (Barba et al., 2016; Sprengers et al., 2017). Multiple lines of evidence suggest that TLE involves a combination of abnormalities in excitatory ion channels, neuroinflammation, oxidative stress, synaptic dysfunction, and structural alterations in the hippocampus and surrounding temporal lobe regions (Englot et al., 2020; Manna et al., 2022). Among them, oxidative stress and inflammation are important links in the pathogenesis of TLE (Maes et al., 2020; Wang et al., 2019). Despite some progress, the precise cellular and molecular mechanisms underlying TLE pathogenesis remain incompletely understood. Therefore, understanding the underlying mechanisms of TLE is crucial for enhancing our comprehension of this intricate neurological condition.

Long noncoding RNAs (lncRNAs) are a class of RNA molecules longer than 200 nucleotides in length and play key roles in the progression of a variety of neurological disorders, such as Alzheimer's disease (AD), Parkinson's disease, ischemic stroke, intracerebral hemorrhage, and TBI (Chen et al., 2021; Ghafouri‐Fard et al., 2022; Kohansal et al., 2024; Li & Wang, 2023). Emerging evidence indicates that lncRNAs play diverse roles in TLE, including modulating neuronal and glial cell functions, regulating apoptosis, affecting synaptic plasticity, and influencing inflammation and oxidative stress responses (Han et al., 2020; Li et al., 2021; Manna et al., 2022; Yazarlou et al., 2024). LncRNA ZFAS1 has been found to play a role in the progression of epilepsy by acting as a sponge for miR‐15a‐5p (Wang et al., 2022). LncRNA ZNF883 mediates the development of epilepsy by promoting inflammation and apoptosis through up‐regulating USP47 by sponging miR‐138‐5p (Gong et al., 2022). A recent study reveals that the level of ILF3‐AS1 is increased in the hippocampus and serum of epilepsy, and upregulation of ILF3‐AS1 level leads to inflammation in astrocytes, which is involved in the development of epilepsy (Cai et al., 2020). However, the functional role of ILF3‐AS1 in TLE has not been studied yet. Hence, the purpose of this work was to explore the ILF3‐AS1‐implicated mechanism in TLE.

MicroRNAs (miRNAs) are small, non‐coding RNA molecules that negatively regulate gene expression by targeting messenger RNAs (mRNAs), playing a fundamental role in various biological processes and diseases, including TLE (Ma, 2018; Martinez & Peplow, 2023). A recent study reveals that miR‐504‐3p expression level is reduced in the brains of patients with AD, and miR‐504‐3p mimics dramatically alleviates tau‐related pathologies (Chen et al., 2022). However, the role of miR‐504‐3p in TLE has not been reported. Notably, one of the most widely recognized mechanisms by which lncRNAs regulate miRNAs is by acting as a “miRNA sponge” (Thomson & Dinger, 2016). This “sponging” effect can inhibit miRNA‐mediated mRNA degradation or translational repression. Using the bioinformatics analysis, we found that ILF3‐AS1 might bind to miR‐504‐3p to regulate high mobile group box 1 (HMGB1) expression, which is the central component of the inflammatory response (Yang et al., 2020). Elevated levels of HMGB1 have been detected after TBI and TLE, and implicated in the progression of secondary brain injury by promoting inflammation and cell death (Chen et al., 2018; Gao et al., 2023), suggesting that HMGB1 contributes to neuronal injury in TLE.

Hence, this study aimed to explore whether ILF3‐AS1 aggravates Mg^2+^‐free‐induced hippocampal neuron injury, an in vitro model of TLE (Busingye et al., 2016; Chen et al., 2021; Zhao et al., 2020), by suppressing oxidative stress and inflammatory response via targeting the miR‐504‐3p/HMGB1 axis.

## 2 MATERIALS AND METHODS

### 2.1 Cell culture and in vitro TLE model

Primary human hippocampal neurons (Sciencell; catalog: #1540) were cultured in neuronal medium (Sciencell; catalog: #1521) at 37°C in an atmosphere containing 5% CO_2_. To simulate TLE in vitro, hippocampal neurons were exposed to Mg^2+^‐free medium for a duration of 3 h and then exchanged for maintenance medium, as has been described in previous studies (Chen et al., 2021; Zhao et al., 2020). Hippocampal neurons grown in the normal culture medium were used as the control.

### 2.2 Quantitative real‐time polymerase chain reaction (qRT‐PCR)

Total RNA was isolated from hippocampal neurons using Trizol reagent (Invitrogen). First‐single‐strand cDNA was synthesized with a PrimeScript™ RT Reagent Kit. Real‐time PCR was conducted employing the SYBR Green PCR Mix Kit (Takara). GAPDH or U6 was used as the internal control. The relative RNA expression levels were calculated with the method of 2^−∆∆Ct^. The primers employed in this study were as follows:

lncRNA ILF3‐AS1 forward: 5′‐GCTTTGACGCATGTGTACCT‐3ʹ
lncRNA ILF3‐AS1 reverse 5ʹ‐CGAAAGAACCCAAGAACCCG‐3ʹ
miR‐504‐3p forward: 5′‐GGGAGAGCAGGGCAG‐3′
miR‐504‐3p reverse: 5′‐GTCCAGTTTTTTTTTTTTTTTGAAACC‐3′
U6 forward: 5′‐CTCGCTTCGGCAGCACA‐3′
U6 reverse 5′‐AACGCTTCACGAATTTGCGT‐3′
HMGB1 forward: 5′‐TATGGCAAAAGCGGACAAGG‐3′
HMGB1 reverse: 5′‐CTTCGCAACATCACCAATGGA‐3′
GAPDH forward 5′‐GGAGCGAGATCCCTCCAAAAT‐3′
GAPDH reverse 5′‐GGCTGTTGTCATACTTCTCATGG’.

### 2.3 Cell transfection

For functional analysis, small interfering RNAs (siRNAs) targeting ILF3‐AS1 (si‐ILF3‐AS1) or ILF3‐AS1 overexpression lentivirus vector (ILF3‐AS1) were used for silencing the expression of ILF3‐AS1 or promoting the expression of ILF3‐AS1, with siRNA random sequence (si‐NC) or empty lentivirus vector (pcDNA) as a negative control (all from GenePharma). MiR‐504‐3p mimics or miR‐504‐3p inhibitors were used for miR‐504‐3p overexpression or inhibition (GenePharma), with mimics negative control (mimics‐NC) or inhibitor negative control (inhibitor‐NC). According to the manufacturer's instructions, hippocampal neurons were transfected using Lipofectamine 3000 (Invitrogen) at 100 nM/well concentrations in Opti‐MEM (Gibco) for 6 h. Then, hippocampal neurons were washed twice with PBS and added to the normal culture for 2 days. The efficiency of knockdown or overexpression was determined by QRT‐qPCR 48 h after transfection.

### 2.4 Bioinformatics analysis

Bioinformatics analysis was used to predict the potential targets of ILF3‐AS1 and miR‐504‐3p, as well as the potential targets of miR‐504‐3p and HMGB1. In brief, the target miRNAs of ILF3‐AS1 were analyzed by TargetScan (https://www.targetscan.org/vert_80/) and Starbase (https://rnasysu.com/encori/). The target mRNAs of miR‐504‐3p were also analyzed by Starbase.

### 2.5 Dual‐luciferase reporter assay

To validate the interaction between ILF3‐AS1 or HMGB1 and miR‐504‐3p, both the wild‐type (WT) and the mutant‐type (MUT) binding regions of ILF3‐AS1 and HMGB1 sequence were cloned into pGL3.Basic vector (Promega), named ILF3‐AS1‐WT, ILF3‐AS1‐MUT, HMGB1‐WT, and HMGB1‐MUT. To validate the interaction between ILF3‐AS1 and miR‐504‐3p, human hippocampal neurons were then transfected with ILF3‐AS1‐WT or ILF3‐AS1‐MUT together with miR‐504‐3p mimics or mimics‐NC using Lipofectamine 3000 (Invitrogen). To validate the interaction between HMGB1 and miR‐504‐3p, human hippocampal neurons were then transfected with HMGB1‐WT or HMGB1‐MUT together with miR‐504‐3p mimics or mimics‐NC using Lipofectamine 3000. At 48 h after transfection, the luciferase activity was assessed using the Dual‐Luciferase Reporter Assay System (Promega) according to the manufacturer's protocol.

### 2.6 Cell counting kit‐8 (CCK‐8) assay

Human hippocampal neurons were seeded into 96‐well culture plates at a density of 1 × 10^4^ cells/cm^2^. Following treatment, cells were incubated with CCK‐8 reagent (Dojindo Molecular Technologies; 10 μL) at 37°C for 3 h. Optical density values were measured at the wavelength of 560 nm using a microplate reader (Thermo, Multiskan MK3).

### 2.7 Lactate dehydrogenase (LDH) detection

LDH is a soluble cytosolic enzyme that is released into the extracellular medium upon damage to the plasma membrane. After treatment, the culture medium from human hippocampal neurons was harvested and LDH release was detected using an LDH cytotoxicity kit (Abcam). The absorbance at 450 nm was determined using a microplate reader (Thermo, Multiskan MK3).

### 2.8 Intracellular reactive oxygen species (ROS), malondialdehyde (MDA), and superoxide dismutase (SOD) levels detection

To detect ROS generation, treated human hippocampal neurons were collected and incubated with DCFH‐DA (Beyotime Biotechnology) at 37°C for 20 min in the dark. The fluorescent images were observed using a fluorescent microscope (Olympus) and the fluorescence intensity was calculated. The level of MDA, an indicator of lipid peroxidation, was determined using a lipid peroxidation assay kit (Solarbio). Meanwhile, SOD activity was assessed using the Total SOD Assay Kit (Beyotime Biotechnology), in accordance with the manufacturer's protocol.

### 2.9 Enzyme‐linked immunosorbent assay (ELISA)

The levels of Interleukin‐6 (IL‐6), IL‐1β, and tumor necrosis factor‐alpha (TNF‐α) in cell culture supernatants were measured using ELISA (Nanjing Jiancheng) following the manufacturer's instructions. The absorbance was tested using a microplate reader (Thermo, Multiskan MK3).

### 2.10 Western blotting analysis

Proteins were extracted from human hippocampal neurons using Lysis Buffer (Beyotime Biotechnology) with 1% v/v phenylmethylsulfonyl fluoride (Beyotime Biotechnology). A total of 30 μg protein samples were separated by sodium dodecyl sulfate–polyacrylamide gel electrophoresis and transferred into polyvinylidene difluoride (Millipore) membranes. After blocking with 5% nonfat dry milk for 2 h at room temperature, the membrane was incubated with the primary antibodies against HMGB1 (1:1000; Abcam) or GAPDH (1: 2000; Abcam) at 4 °C overnight. Subsequently, the membranes were incubated with corresponding horseradish peroxidase‐conjugated secondary antibodies for 1 h at room temperature. Then, the membranes were visualized using the ECL‐Western blotting system (BioRad), and the band intensities were quantified using ImageJ software (National Institutes of Health).

### 2.11 Statistical analysis

Data are expressed as the mean ± standard deviation (SD). Statistical analyses were carried out using SPSS version 17.0 (IBM). The discrepancy between groups was performed by the Student's *t*‐test or ANOVA together with Turkey's test. A *p* < .05 was considered statistically significant.

## 3 RESULTS

### 3.1 ILF3‐AS1 was elevated in Mg^2+^‐free‐treated hippocampal neurons and ILF3‐AS1 knockdown attenuates the hippocampal neurons injuries

**FIGURE 1:**
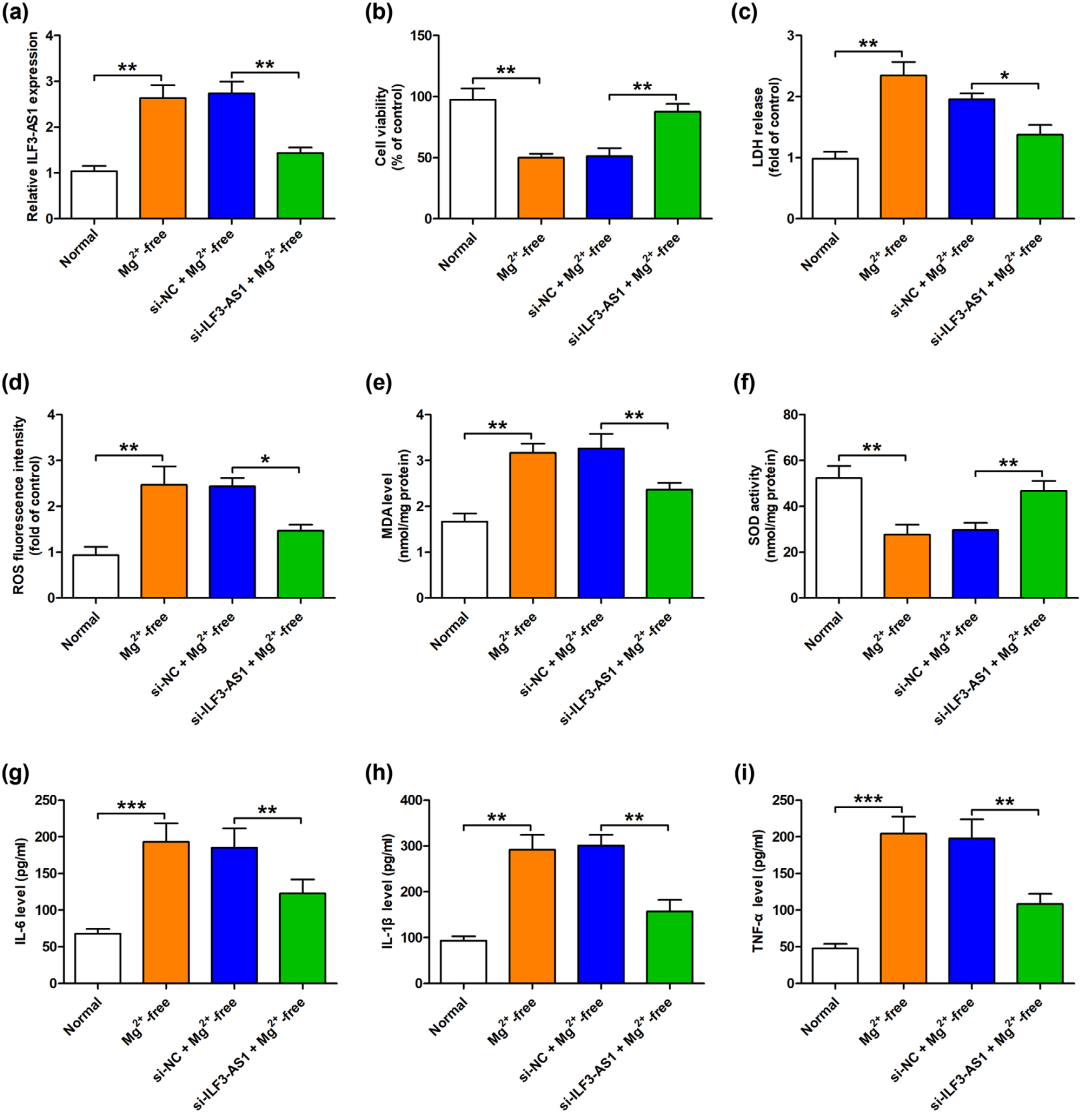
ILF3‐AS1 knockdown attenuated Mg^2+^‐free‐induced hippocampal neurons injuries. (a) The level of ILF3‐AS1 in the Mg^2+^‐free‐treated hippocampal neurons were measured by Quantitative real‐time polymerase chain reaction (qRT‐qPCR). (b) Cell viability was detected by cell counting kit‐8 assay. (c) Lactate dehydrogenase (LDH) release was assayed by the LDH cytotoxicity kit. (d) Reactive oxygen species (ROS) generation, (e) malondialdehyde (MDA) content, and (f) superoxide dismutase (SOD) activity were examined by the corresponding kit. (g) Interleukin‐6 (IL‐6), (h) IL‐1β, and (i) tumor necrosis factor‐alpha (TNF‐α) levels were determined by enzyme‐linked immunosorbent assay (ELISA). Experiments were carried out at least in triplicates (^*^ *p *< .05, ^**^ *p *< .01, ^***^ *p *< .001).

First, we examined the expression of ILF3‐AS1 in a cell model of TLE by qRT‐PCR analysis. We found that the expression of ILF3‐AS1 was significantly increased in Mg^2+^‐free‐exposed hippocampal neurons compared to the normal group (Figure 1a). To verify the role of ILF3‐AS1 in the development of TLE, hippocampal neurons treated with Mg^2+^‐free were transfected with si‐ILF3‐AS1 or si‐NC. We observed that ILF3‐AS1 knockdown induced by transfection with si‐ILF3‐AS1 significantly recovered cell viability in Mg^2+^‐free‐exposed hippocampal neurons compared to the transfection with si‐NC group (si‐NC) (Figure 1a,b). The upregulation of LDH release in Mg^2+^‐free‐treated hippocampal neurons was also attenuated by ILF3‐AS1 knockdown (Figure 1c). Given that oxidative stress and inflammation are important indicators in the pathogenesis of TLE, we next demonstrate the role of ILF3‐AS1 in Mg^2+^‐free‐induced oxidative stress and inflammatory response. We found that the levels of oxidative stress‐related markers including ROS and MDA were increased in Mg^2+^‐free‐treated hippocampal neurons; after ILF3‐AS1 knockdown, the levels of ROS and MDA in Mg^2+^‐free‐treated hippocampal neurons were decreased relative to the si‐NC group (Figure 1d,e). In addition, the reduction of SOD activity in Mg^2+^‐free‐treated hippocampal neurons was reversed by ILF3‐AS1 knockdown in comparison with the si‐NC group (Figure 1f). Furthermore, ELISA results revealed that the levels of inflammatory cytokines IL‐1β, IL‐6, and TNF‐α were consequently upregulated after Mg^2+^‐free induction, while ILF3‐AS1 knockdown significantly blocked these effects compared to the si‐NC group (Figure 1g–i). Therefore, our findings indicated that ILF3‐AS1 greatly contributed to Mg^2+^‐free‐induced hippocampal neuron injuries by promoting oxidative stress and inflammatory response.

### 3.2 MiR‐504‐3p was a target of ILF3‐AS1

**FIGURE 2:**
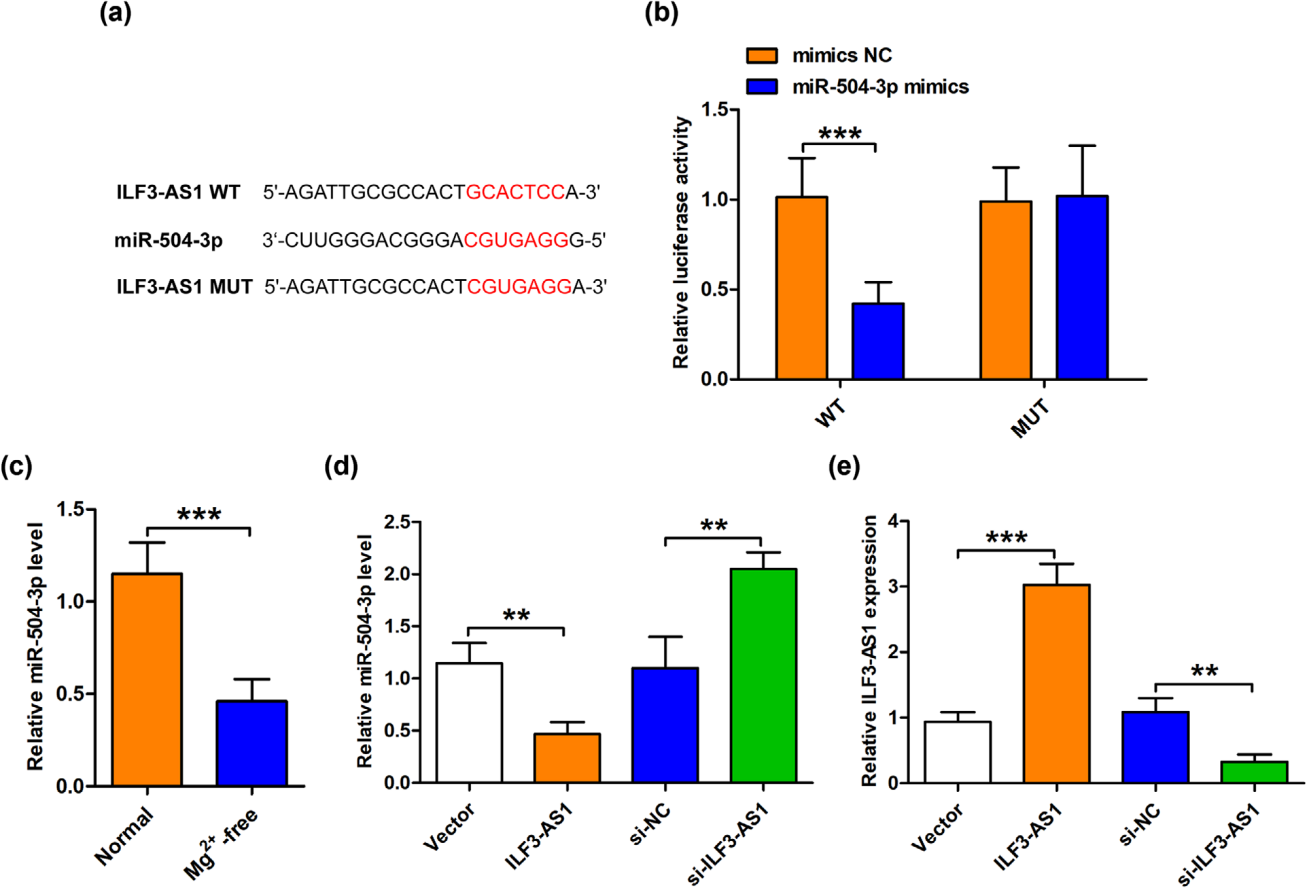
ILF3‐AS1 targeted and negatively regulated miR‐504‐3p. (a) Schematic diagram shows the predicted wild‐type (WT) and binding mutant (MUT) of ILF3‐AS1 for miR‐504‐3p. The targeted relationship between ILF3‐AS1 and miR‐504‐3p was verified by Dual‐luciferase reporter gene analysis. (b) The level of miR‐504‐3p in the normal and Mg^2+^‐free‐treated hippocampal neurons was detected by QRT‐qPCR analysis. (C,D) The levels of miR‐504‐3p and ILF3‐AS1 in the control, ILF3‐AS1‐silenced, and ILF3‐AS1‐overexpressing cells were measured by QRT‐qPCR analysis. Experiments were carried out at least in triplicates (^**^ *p *< .01 and ^***^ *p *< .001).

Next, we explored the exact mechanism of ILF3‐AS1 function in TLE. Using the bioinformatics analysis, we found that miR‐504‐3p was a potential target gene of ILF3‐AS1, and the potential binding site between miR‐504‐3p and ILF3‐AS1 is shown in Figure 2a. To confirm the interaction, a dual‐luciferase reporter assay was carried out and the results revealed that miR‐504‐3p mimics significantly decreased the luciferase activity of ILF3‐AS1‐WT but not ILF3‐AS1‐MUT (Figure 2b). Subsequently, whether Mg^2+^‐free‐affected miR‐504‐3p level was detected in human hippocampal neurons. QRT‐qPCR results showed that Mg^2+^‐free dramatically reduced the level of miR‐504‐3p in hippocampal neurons compared with the normal group (Figure 2c). Furthermore, we assessed the effects of ILF3‐AS1 overexpression and knockdown on miR‐504‐3p expression. QRT‐qPCR results revealed that ILF3‐AS1 overexpression induced by transfection with ILF3‐AS1 reduced the expression of miR‐504‐3p, while ILF3‐AS1 knockdown induced by transfection with si‐ILF3‐AS1 increased the expression of miR‐504‐3p (Figure 2d,e). Collectively, these results confirmed that miR‐504‐3p was a target of ILF3‐AS1 and negatively regulated by ILF3‐AS1.

### 3.3 ILF3‐AS1 knockdown alleviates Mg^2+^‐free‐induced hippocampal neurons injuries via targeting miR‐504‐3p

**FIGURE 3:**
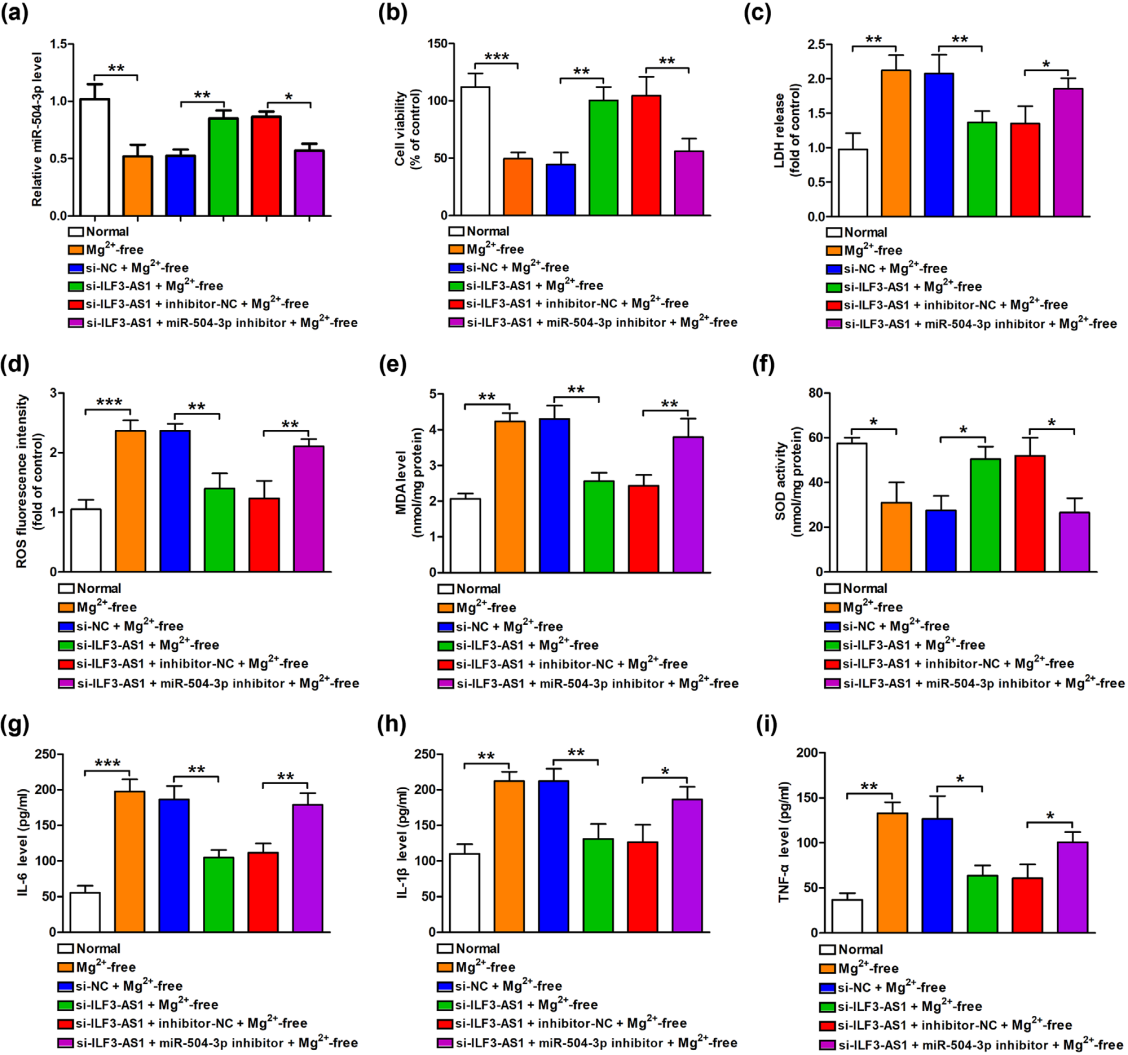
ILF3‐AS1 knockdown mitigated Mg^2+^‐free‐induced hippocampal neurons injuries by targeting miR‐504‐3p. (a) The expression of miR‐504‐3p in the hippocampal neurons co‐transfected with si‐ILF3‐AS1 and miR‐504‐3p inhibitor were measured by QRT‐qPCR. (b) Cell viability was detected by cell counting kit‐8. (c) Lactate dehydrogenase (LDH) release was assayed by the LDH cytotoxicity kit. (d) Reactive oxygen species (ROS) generation, (e) malondialdehyde (MDA) content, and (f) superoxide dismutase (SOD) activity were examined by the corresponding kit. (g) Interleukin‐6 (IL‐6), (H) IL‐1β, and (I) tumor necrosis factor‐alpha (TNF‐α) levels were determined by enzyme‐linked immunosorbent assay (ELISA). Experiments were carried out at least in triplicates (^*^ *p *< .05, ^**^ *p *< .01, ^***^ *p *< .001).

To confirm the role of miR‐504‐3p in the protection of ILF3‐AS1 knockdown against Mg^2+^‐free‐induced neuron injuries, hippocampal neurons were co‐transfected with a miR‐504‐3p inhibitor and si‐ILF3‐AS1. ILF3‐AS1 knockdown‐induced increased miR‐504‐3p levels were significantly blocked by the miR‐504‐3p inhibitor (Figure 3a). CCK‐8 assay results showed that ILF3‐AS1 knockdown‐induced up‐regulation of cell viability (Figure 3b) and down‐regulation of LDH release (Figure 3c) in Mg^2+^‐free‐treated hippocampal neurons were also abrogated by miR‐504‐3p inhibitor. Moreover, ILF3‐AS1 knockdown‐suppressed oxidative stress in Mg^2+^‐free‐treated hippocampal neurons was blocked by miR‐504‐3p inhibitor, as evidenced by the increases in ROS and MDA levels and the decrease in SOD activity (Figure 3d–f). The inflammatory response in Mg^2+^‐free‐exposed hippocampal neurons was inhibited by ILF3‐AS1 knockdown, which was also mitigated by the miR‐504‐3p inhibitor (Figure 3g–i). Taken together, these results indicated that ILF3‐AS1 binding to miR‐504‐3p contributed to hippocampal neuron injuries, oxidative stress, and inflammation during TLE in vitro.

### 3.4 HMGB1 was a direct target of miR‐504‐3p

**FIGURE 4:**
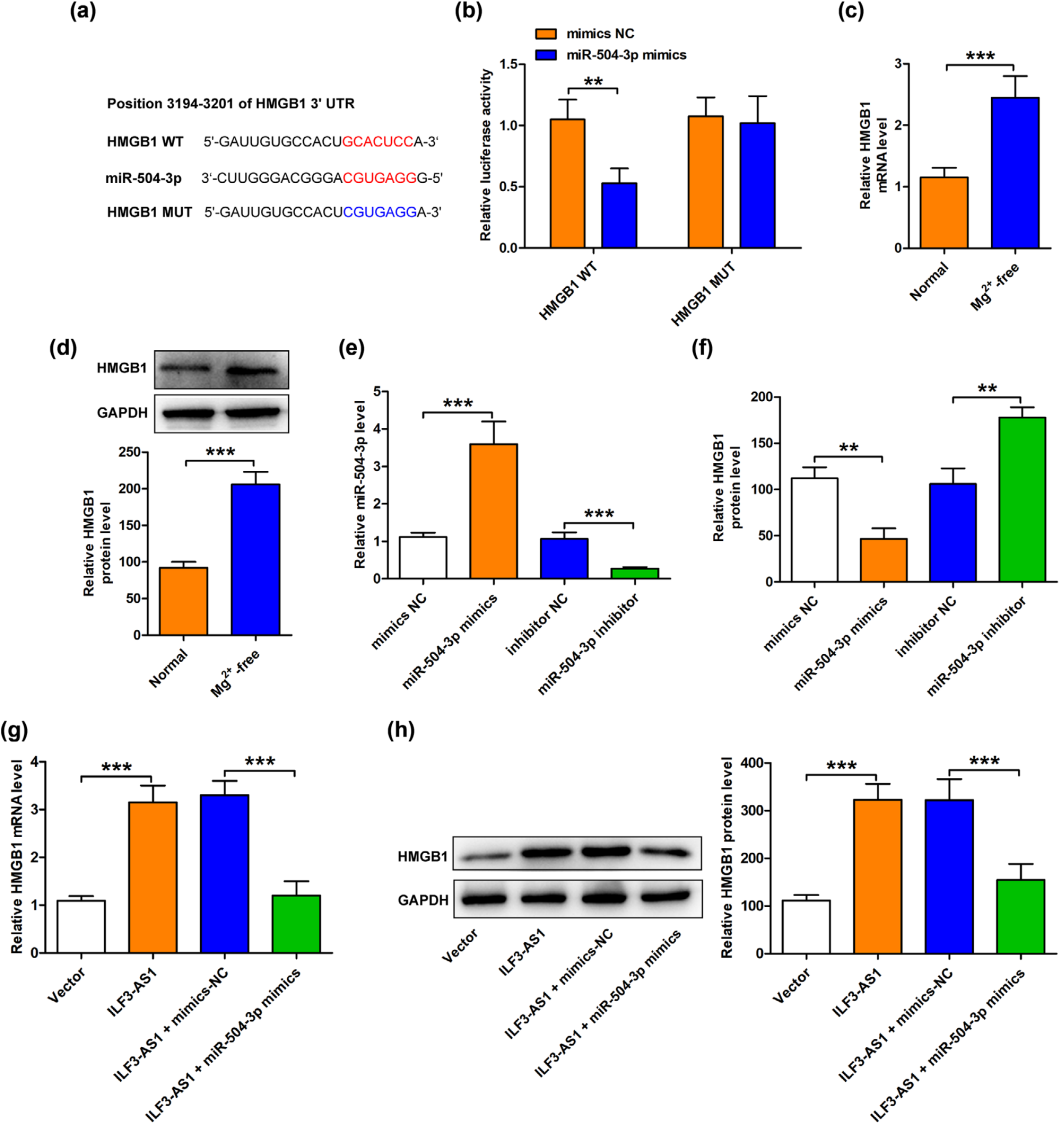
High mobile group box 1 (HMGB1) was a target of miR‐504‐3p and ILF3‐AS1 promoted the protein expression of HMGB1 by targeting miR‐504‐3p. (a) The binding sites between miR‐504‐3p and HMGB1 were predicted by StarBase. (b) The interaction between miR‐504‐3p and HMGB1 was examined by Dual‐luciferase reporter assay. (c) The expression of HMGB1 in Mg^2+^‐free‐induced hippocampal neurons was measured by Western blot analysis. (d) The efficiency of miR‐504‐3p overexpression or knockdown was confirmed by quantitative real‐time polymerase chain reaction (qRT‐PCR). The mRNA (e) and protein expression (f) levels of HMGB1 in miR‐504‐3p overexpression or knockdown‐induced human hippocampal neurons were detected by the qRT‐PCR assay and Western blot analysis, respectively. The mRNA (g) and protein expression (h) levels of HMGB1 in cotransfection of si‐ILF3‐AS1 + anti‐ miR‐504‐3p were examined by the qRT‐PCR assay and Western blot analysis. Experiments were carried out at least in triplicates (^*^ *p *< .05, ^**^ *p *< .01, ^***^ *p *< .001).

Then, we analyzed the potential target mRNAs of miR‐504‐3p using Target scan software/Starbase and found that there were several binding sites between miR‐504‐3p and HMGB1 (Figure 4a). Dual‐luciferase reporter assay was performed to confirm the interaction between miR‐504‐3p and HMGB1 and results showed that miR‐504‐3p mimics significantly attenuated the luciferase activity of HMGB1‐WT but not HMGB1‐MUT (Figure 4b). Notably, both mRNA and protein expression levels of HMGB1 were increased in Mg^2+^‐free‐treated hippocampal neurons (Figure 4c,d). Furthermore, promoting miR‐504‐3p level induced by miR‐504‐3p mimics greatly decreased the mRNA level of HMGB1, while inhibiting miR‐504‐3p level induced by miR‐504‐3p inhibitor increased the mRNA level of HMGB1 (Figure 4e,f). Similarly, transfection with si‐ILF3‐AS1 reduced the mRNA and protein expression levels of HMGB1, which were restored by the miR‐504‐3p inhibitor (Figure 4g,h). The data implied that miR‐504‐3p directly binds with HMGB1 and ILF3‐AS1 regulated the expression of HMGB1 by sponging miR‐504‐3p.

### 3.5 HMGB1 overexpression blocked the protective effects of ILF3‐AS1 knockdown on Mg^2+^‐free‐induced hippocampal neurons injuries

**FIGURE 5:**
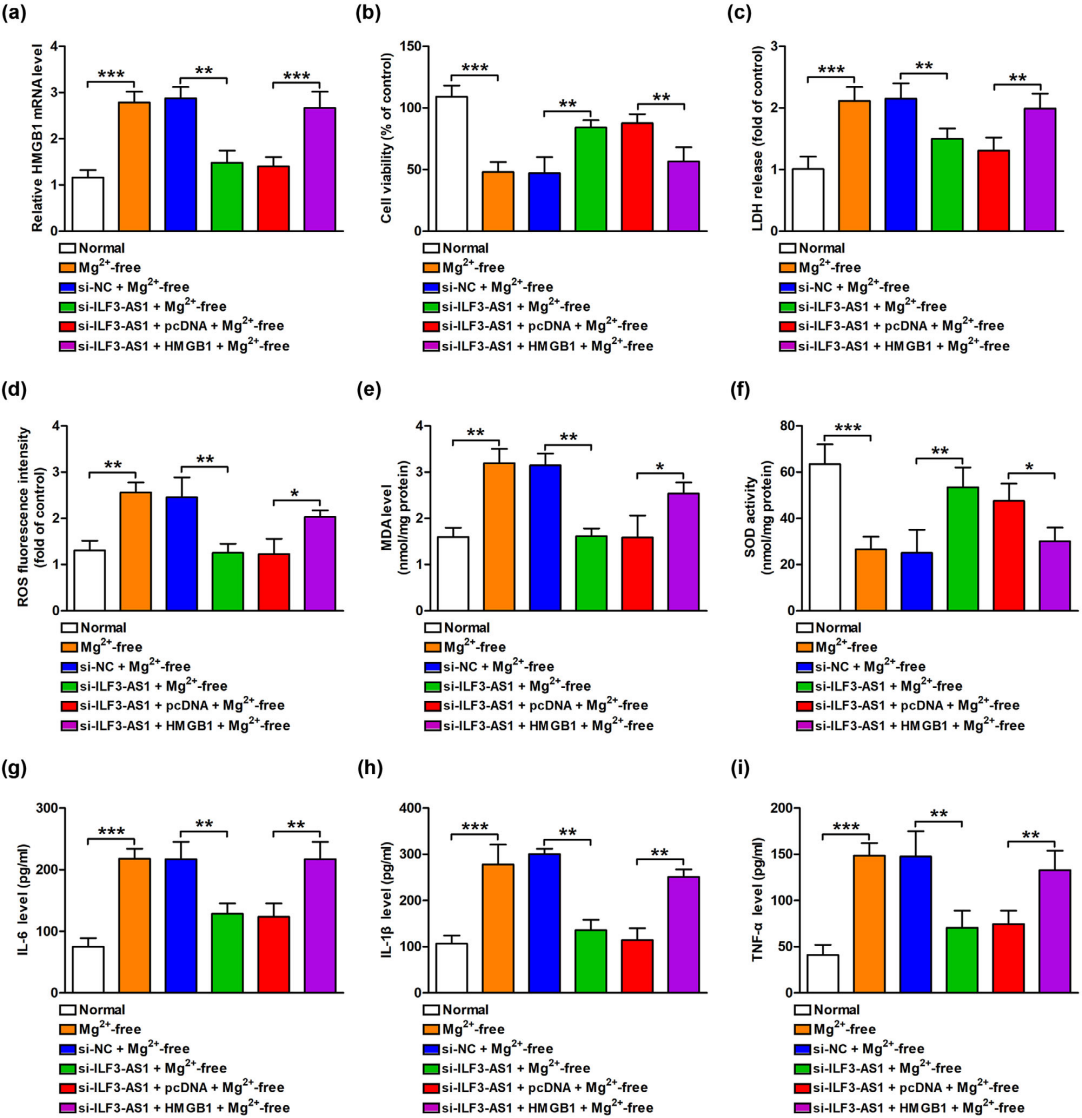
ILF3‐AS1 knockdown alleviated hippocampal neuron injury by regulating high mobile group box 1 (HMGB1). (a) The mRNA level of HMGB1 in the hippocampal neurons co‐transfected with si‐ILF3‐AS1 and HMGB1 were measured by QRT‐qPCR assay. (b) Cell viability was detected by cell counting kit‐8 (CCK‐8) assay. (c) Lactate dehydrogenase (LDH release was assayed by LDH cytotoxicity kit. (d) Reactive oxygen species (ROS) generation, (e) malondialdehyde (MDA) content, and (f) superoxide dismutase (SOD) activity were examined by the corresponding kit. (g) Interleukin‐6 (IL‐6), (H) IL‐1β, and (i) tumor necrosis factor‐alpha (TNF‐α) levels were determined by enzyme‐linked immunosorbent assay (ELISA). Experiments were carried out at least in triplicates (^*^ *p *< .05, ^**^ *p *< .01, ^***^ *p *< .001).

To verify the role of HMGB1 in the mechanism of ILF3‐AS1 in regulating TLE, hippocampal neurons were transfected with ILF3‐AS1‐siRNA combined with HMGB1. The inhibition of ILF3‐AS1 knockdown on the mRNA level of HMGB1 was rescued by HMGB1 overexpression (Figure 5a). In addition, the upregulation of cell viability (Figure 5b) and the down‐regulation of LDH release (Figure 5c) induced by ILF3‐AS1 knockdown were blocked by HMGB1 overexpression in Mg^2+^‐free‐induced hippocampal neurons. Furthermore, ILF3‐AS1 knockdown‐induced inhibition of oxidative stress in Mg^2+^‐free‐induced hippocampal neurons was also attenuated by Mg^2+^‐free‐induced hippocampal neurons, as evidenced by the increases in ROS and MDA contents and a decrease in SOD activity (Figure 5d–f). Moreover, the inhibition of ILF3‐AS1 knockdown on inflammatory response was notably reversed by HMGB1 overexpression in Mg^2+^‐free‐induced hippocampal neurons (Figure 5g–i). Therefore, these data demonstrated that ILF3‐AS1 promoted Mg^2+^‐free‐induced hippocampal neuron injuries by regulating HMGB1.

## 4 DISCUSSION

**FIGURE 6:**
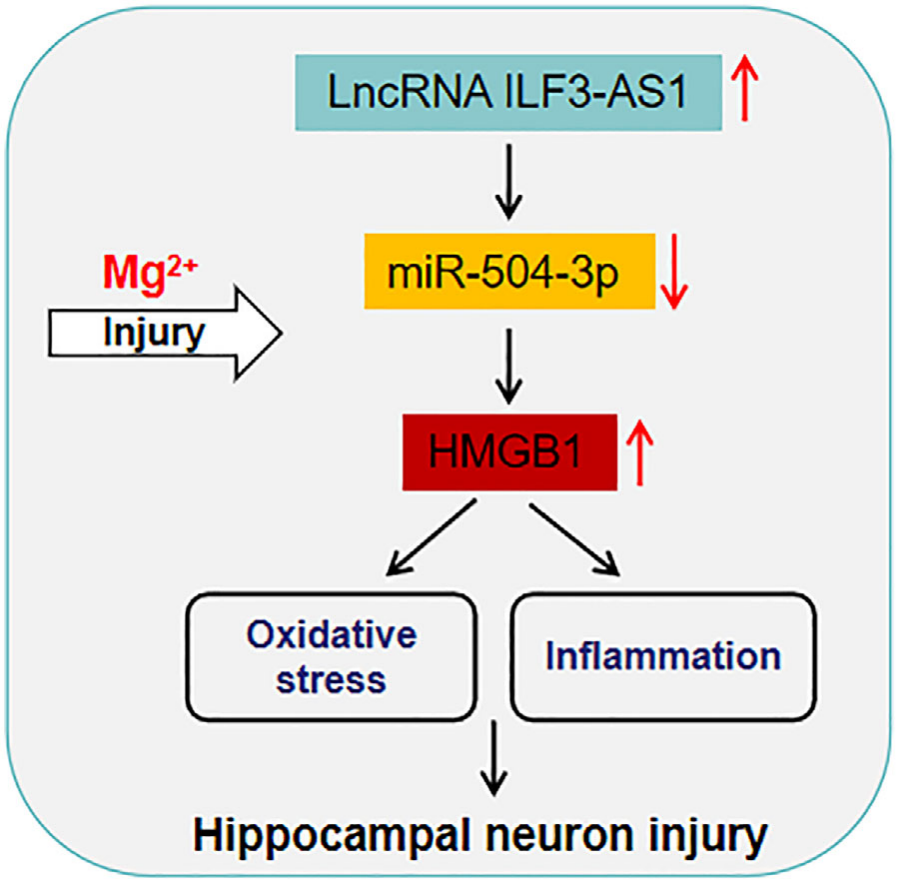
The summary diagram of this study. Our findings indicate that ILF3‐AS1 contributes to hippocampal neuron injuries following temporal lobe epilepsy (TLE) by inhibiting neuronal oxidative stress and inflammation through the miR‐504‐3p/HMGB1 pathway in vitro.

TLE is a chronic neurological disorder characterized by recurrent seizures and associated with hippocampal dysfunction. Emerging evidence suggests that long non‐coding RNAs (lncRNAs) play crucial roles in the pathogenesis of TLE. Recently, it is reported that LncRNA ILF3‐AS1 is implicated in the pathogenesis of epilepsy; however, the functional roles of ILF3‐AS1 in TLE and the underlying mechanisms remain unclear. This study revealed that Asprosin mediates hippocampal neuron injuries after TLE in vitro by inhibiting oxidative stress and inflammation through miR‐504‐3p/HMGB1 axis (Figure 6).

Emerging evidence highlights the significant involvement of lncRNAs in the pathogenesis of TLE, which are involved in diverse biological processes, such as neuronal development, synaptic plasticity, inflammation, oxidative stress, and apoptosis (Jang et al., 2018; Manna et al., 2022; Wan & Yang, 2020). For instance, HOTAIR is increased in both animal models and human TLE patients and has been shown to influence neuroinflammatory processes and modulate gene expression patterns associated with TLE pathogenesis (Cheng et al., 2021). NEAT1 has been associated with neuroinflammation, neuronal excitability, and seizure susceptibility, suggesting its potential involvement in TLE pathophysiology (Wan & Yang, 2020). Recently, altered expression of ILF3‐AS1 also has been observed in TLE (Cai et al., 2020). ILF3‐AS1 level is elevated in the hippocampus and serum of TLE patients; And ILF3‐AS1 promotes inflammatory cytokines. However, the roles and underlying mechanisms of ILF3‐AS1 in TLE remain further investigation. In our study, ILF3‐AS1 was found to be significantly increased in Mg^2+^‐free‐treated human hippocampal neurons, an in vitro cell model of TLE (Geng et al., 2018; Yuan et al., 2016), and knockdown of ILF3‐AS1 ameliorated Mg^2+^‐free‐induced hippocampal neurons injury, oxidative stress and inflammatory response as indicated by the increase in cell viability, the decline in ROS and MDA level as well as the significant increase in SOD activity, and the increases in IL‐6, IL‐1β, and TNF‐a levels, suggesting that ILF3‐AS1 contributes to the pathogenesis of TLE in vitro via promoting oxidative stress and inflammatory response. These discoveries indicate that ILF3‐AS1 may be an effective biomarker or potential target for treating TLE.

ILF3‐AS1 has been identified as a ceRNA that competes with miRNAs for binding sites, thereby modulating the activity of miRNAs and indirectly regulating the expression of their downstream target genes. For example, ILF3‐AS1 has been shown to interact with miR‐200b‐3p, leading to the repression of ZEB1 expression and influencing cell proliferation and metastasis in gastric cancer. During the process of TLE, ILF3‐AS1 induces the expression of inflammatory cytokines by targeting miR‐212 (Cai et al., 2020). Hence, to further understand the molecular mechanism of ILF3‐AS1 in TLE, target miRNA was predicted and verified through a dual‐luciferase reporter assay. Mechanically, we validated that miR‐504‐3p was a target of ILF3‐AS1 and negatively regulated by ILF3‐AS1. MiR‐504‐3p inhibitors suppressed si‐ILF3‐AS1‐induced the protection against Mg^2+^‐free‐induced hippocampal neuron injuries, oxidative stress, and inflammatory response. Previous research has revealed that miR‐504‐3p expression is reduced in the brains of Alzheimer's disease (AD) patients, which may be an early event in the process of AD (Chen et al., 2022; Improta‐Caria et al., 2020). Increasing evidence shows a close association between the development of AD and epilepsy (Fang et al., 2023; Li et al., 2020). These evidences indicate that miR‐504‐3p may participate in the role of ILF3‐AS1 in TLE. Our results further found that miR‐504‐3p inhibitors suppressed si‐ILF3‐AS1‐induced the protection against Mg^2+^‐free‐induced hippocampal neuron injuries, oxidative stress, and inflammatory response. MiR‐504‐3p inhibitors suppressed si‐ILF3‐AS1‐induced the protection against Mg^2+^‐free‐induced hippocampal neuron injuries, oxidative stress, and inflammatory response. In TLE, increased oxidative stress and inflammation are key contributors to neuronal damage and seizure progression (Bernardino et al., 2005; Puttachary et al., 2015). In line with these data, our results discovered that ILF3‐AS1 binding to miR‐504‐3p promotes oxidative stress and inflammation, thereby leading to hippocampal neuron injuries in TLE.

Using the starBase database, we found that one of the target genes of miR‐504‐3p is High Mobility Group Box 1 (HMGB1), which is a pro‐inflammatory cytokine involved in the activation of immune responses and inflammatory signaling pathways (Andersson et al., 2018; Yuan et al., 2016). In TLE, miRNA‐29a targeting HMGB1 expression leads to inflammation and neuronal damage (Wu et al., 2021). In addition, inhibition of HMGB1‐mediated oxidative stress and inflammation provides a neuroprotective effect in TBI mice (Wang et al., 2020). Consistent with these studies, our results revealed that HMGB1 was also a target of miR‐504‐3p. Notably, ILF3‐AS1 regulated the expression of HMGB1 by sponging miR‐504‐3p. Moreover, our rescue experiment confirmed that HMGB1 overexpression could partially reverse the protective effects of ILF3‐AS1 knockdown on hippocampal neuron injuries, oxidative stress, and inflammatory response.

However, this study has several limitations. First, while the study provides detailed insights into the role of ILF3‐AS1 in a cellular model of TLE, it lacks in vivo validation. Further testing in *vivo* animal models could help confirm the relevance and potential therapeutic implications of these findings. Second, the relationship between ILF3‐AS1 and other potential targeting miRNAs needed more attentions and researches. In addition, since the study was conducted using human hippocampal neurons in vitro, the generalizability of the results to other types of neurons or to clinical TLE remains uncertain. Studies involving different neuronal types and clinical samples could validate the findings more broadly.

Taken together, these findings further demonstrate that ILF3‐AS1 contributes to hippocampal neuron injuries following TLE by inhibiting neuronal oxidative stress and inflammation through the miR‐504‐3p/HMGB1 pathway in vitro. Our data supported that ILF3‐AS1 participates in the development of TLE and may provide a new regulatory mechanism and alternative avenues for TLE progression.

## AUTHOR CONTRIBUTIONS

**Peipei Gao**: Conceptualization; investigation; data curation; writing—original draft; funding acquisition. **Ying Wu**: Data curation; investigation. **Zhixin Yan**: Writing—original draft; investigation; data curation; writing—review and editing.

## CONFLICT OF INTEREST STATEMENT

All authors declare no conflict of interest.

### PEER REVIEW

The peer review history for this article is available at https://publons.com/publon/10.1002/brb3.3615.

## Data Availability

All data supporting the findings of this study can be requested from the corresponding author.
